# Production of Native Bispecific Antibodies in Rabbits

**DOI:** 10.1371/journal.pone.0010879

**Published:** 2010-06-14

**Authors:** Wei Wang, Ruihuan Xu, Jinming Li

**Affiliations:** 1 Graduate School, Peking Union Medical College, Chinese Academy of Medical Sciences, Beijing, People's Republic of China; 2 National Center for Clinical Laboratories, Beijing Hospital, Beijing, People's Republic of China; Dana-Farber Cancer Institute, United States of America

## Abstract

**Background:**

A natural bispecific antibody, which can be produced by exchanging Fab arms of two IgG4 molecules, was first described in allergic patients receiving therapeutic injections with two distinct allergens. However, no information has been published on the production of natural bispecific antibody in animals. Even more important, establishment of an animal model is a useful approach to investigate and characterize the naturally occurring antibody.

**Methodology/Principal Findings:**

We demonstrated that a natural bispecific antibody can also be generated in New Zealand white rabbits by immunization with synthesized conjugates. These antibodies showed bispecificity to the components that were simultaneously used to immunize the animals. We observed a trend in our test animals that female rabbits exhibited stronger bispecific antibody responses than males. The bispecific antibody was monomeric and primarily belonged to immunoglobulin (Ig) G. Moreover, bispecific antibodies were demonstrated by mixing 2 purified monospecific antibodies *in vivo* and *in vitro*.

**Conclusions/Significance:**

Our results extend the context of natural bispecific antibodies on the basis of bispecific IgG4, and may provide insights into the exploration of native bispecific antibodies in immunological diseases.

## Introduction

Antibodies exist as one or more copies of a Y-shaped unit. Each Y-shaped unit contains 4 polypeptides, 2 identical copies of a polypeptide known as the heavy chain and 2 identical copies of a polypeptide called the light chain. Immunoglobulin G (IgG) antibodies contain only 1 structural Y-shaped unit and are also the most abundant in serum. The enzyme papain cleaves an IgG molecule into 3 active fragments. Two of these are identical antigen-binding fragments (Fab), which form the arms of the Y and make an IgG molecule bivalent. The third fragment, which forms the base of the Y, is called Fc fragment because it is readily crystallized. This region is important in mediating the effector functions of the antibody in immune responses [1].

Bispecific antibody, as its name indicates, usually consists of 2 distinct Fab arms, by which it is capable of simultaneously binding two different antigens. This characteristic confers bispecific antibody immense potential for a wide range of clinical applications as targeting agents for immunodiagnosis and therapy. It is believed that bispecific antibody usually do not occur naturally but are constructed artificially by recombinant DNA or cell-fusion technologies [2]. However, a class of natural bispecific antibody was recently obtained from allergic patients receiving therapeutic injections with 2 different allergens during specific immunotherapy [3]. It was indicated that the antibody with bispecificity belonged to the IgG4 subclass. IgG4 antibodies are dynamic molecules that exchange their Fab arms by swapping a heavy chain and attached light chain (half-molecule) with a heavy-light chain pair from another molecule, resulting in bispecific antibodies [3]–[4]. This has been demonstrated in a rhesus monkey model with experimental autoimmune myasthenia gravis. Interestingly, the exchanged IgG4 did not cause disease, instead, it appeared to protect the animal from IgG1-mediated inflammation, probably by displacing the IgG1 and binding monovalently to acetylcholine receptors (AchR) [4]. Nevertheless, the origin of natural bispecific antibodies and their significances remain to be further determined.

In this report, we described our investigations on animal models for the production of natural bispecific antibodies against different synthesized conjugates. Purified bispecific antibodies were obtained and their characteristics were examined.

## Materials and Methods

### Conjugation

Digoxin, horseradish peroxidase (HRP), keyhole limpet hemocyanin (KLH), and bovine serum albumin (BSA) were purchased from Sigma-Aldrich (St. Louis, MO, USA). KLH-dinitrophenyl (KLH-DNP) conjugates were obtained from Merck (Darmstadt, Germany). BSA-DNP was purchased from Biosearch Technologies (Novato, CA).

Digoxin was conjugated to BSA using a previously described method [5]–[6]. The efficiency of conjugation was evaluated spectrophotometrically in 83% H_2_SO_4_ [7]. To establish enzyme-linked immunosorbent assays (ELISA), each of the 4 antigens, BSA, KLH, DNP, and digoxin, was labeled with HRP, using the periodate oxidation method [5]. After purification, the optimal working concentration for each enzyme conjugate was determined by chessboard titrations [8].

### Animal model

24 New Zealand white rabbits were allocated to 6 groups marked as (1) BSA group, (2) Digoxin group, (3) BSA-digoxin group, (4) BSA-DNP group, (5) KLH-DNP group, and (6) BSA-Digoxin/KLH-DNP mixed group. Each group comprised 2 males and 2 female rabbits. Blank sera were obtained before the immunization procedure. For primary immunization, immunogens were dissolved in 0.85% saline at a concentration of 2 mg/mL and emulsified with an equal volume of complete Freund's adjuvant (CFA), which contained approximately 1 mg/mL of killed tubercle bacilli [9]. The rabbits were immunized subcutaneously at multiple sites along the spinal cord. After 3 months, booster injections were administered with incomplete Freund's adjuvant (IFA), at a dose equivalent to half of the primary. The procedure was performed at 2-month intervals for 7 months [10]. Blood was drawn from the marginal ear vein and the titers of antibodies were evaluated.

All rabbits were raised under standardized pathogen-free conditions in the Animal Care Facility at Beijing Hospital. The study protocol for the experimental use of the animals was approved by the Ethics Committee of National Center for Clinical Laboratories.

### Monospecific antibody ELISA and bispecific antibody ELISA

To test the ability of antibodies to crosslink the same antigens, carriers or carrier-hapten conjugates in 0.05 M carbonate buffer (pH 9.6) were coated onto ELISA plates (Costar, USA) at 4°C overnight. The plates were subsequently washed thoroughly with phosphate-buffered saline (PBS), and the unbound sites were blocked with 1% (w/v) gelatin in PBS at 37°C for 2 h. After washing 3 times with PBS containing 0.05% (w/v) Tween-20 (PBST), 100 µL of serially diluted sera were incubated with coated antigens at 37°C for 1 h. Blank sera were tested as controls. Subsequently, the plates were washed as above, and HRP-labeled carriers or haptens, which were the same as those used for the coating, were optimally diluted using 0.2% gelatin in PBST and 100 µL was applied to each well. After incubation at 37°C for 1 h, 100 µL of tetramethyl benzidine (TMB) solution was added to each well. Color development was terminated after incubation at 37°C for 30 min by addition of 50 µL of 2 M H_2_SO_4_, and the optical density (OD) was measured at 450 nm (with 620 nm as a reference) using an ELISA plate reader (Labsystems, Finland).

The procedures for the bispecific antibody ELISA were similar to those described above, with the exception that the detection antigens, which were labeled with HRP, were different from the coating, according to specific situations. Thus, when BSA was the coated antigen, HRP-labeled digoxin was used to detect the bispecific antibody against BSA and digoxin. The others were detected in the same manner. Each serum was detected in duplicate. The end point dilution titers for each antibody were defined as the maximum dilution of serum giving an absorbance reading of 0.1 units over the blank sera wells after subtracting the background caused by enzyme conjugates.

### Bispecific inhibition assay

For inhibition studies, 50 µL of non-labeled digoxin and DNP at different concentrations in PBS were used as inhibitors and mixed with an equal volume of HRP-labeled digoxin and HRP-labeled DNP, respectively. The mixtures were used as a substitute for 100 µL of HRP-labeled antigens in the bispecific antibody ELISA.

### Size-exclusion chromatography

BSA-digoxin immunized serum (200 µL), which was determined to contain bispecific antibodies, was subjected to gel filtration to exclude the involvement of antibody aggregates. Size-exclusion chromatography was performed on a Bio-Gel A1.5m (Bio-Rad Laboratories, USA) column (1.5×45 cm). The column was equilibrated and washed with 0.1 M PBS (pH 7.4). Fractions of 500 µL were collected and analyzed for the bispecificity of the antibodies. Furthermore, the IgG nature of the antibodies was determined by a HRP conjugated goat anti-rabbit IgG antibody (1∶20000, Sigma-Aldrich). Briefly, BSA-digoxin conjugates were coated onto the ELISA plates. After incubation with the sera, the captured rabbit IgG antibodies to BSA-digoxin were detected by the anti-rabbit IgG secondary antibody. As a control, human normal immunoglobulin for intravenous injection (200 µL; Green Cross China Biological Products, Anhui Province, China), which comprises primarily IgG monomers, was fractionated on the same column.

### Pepsin digestion

Pepsin digestion of antiserum was carried out as described previously to exclude IgG Fc-Fc interactions [11]–[14]. Briefly, antiserum containing bispecific antibodies against BSA and digoxin was dialyzed in 0.2 M sodium acetate buffer (pH 4.0) at 4°C overnight. Protein (2 mg/mL; estimated as 1.54×A_280_−0.76×A_260_) was mixed with equal volume of 40 µg/mL of pepsin (Sigma) or acetate buffer. The test samples and controls were incubated at 37°C. The digestion reaction was stopped by raising the pH to pH 8.0 using 2 M Tris base at different time intervals. Products of digestion were analyzed by the bispecific antibody ELISA.

### Purification of bispecific antibodies

Purification of bispecific antibodies against BSA and digoxin was performed using BSA-Sepharose and digoxin-Sepharose. Conjugation of BSA to CNBr-activated Sepharose 4B (GE Healthcare, Amersham Biosciences, Sweden) was conducted according to the manufacturer's instructions. The conjugation of digoxin to ω-aminoalkyl Sepharose (GE Healthcare, Amersham Biosciences, Sweden) was conducted by a previously described method [15].

First, rabbit immunoglobulins were precipitated by 50% ammonium sulfate [16]. The precipitates were redissolved in PBS and fractionated on a protein A (Pharmacia, America) column according to manufacturer's protocol. IgG fractions were collected and pooled, dialyzed against 0.1 M PBS, and concentrated to about 10 mg/mL. BSA- and digoxin-conjugated matrixes were equilibrated with 0.1 M PBS (pH 7.4) for experiments. The immunoglobulin solution was first applied to the digoxin-Sepharose column. The unbound components, primarily monospecific antibodies against BSA (anti-BSA), were collected. The bound antibodies were eluted using 25% methanol (pH 2.5). The eluted fractions were neutralized immediately with 10 mM PB (pH 7.4), pooled, and dialyzed. Then, the eluted portion was loaded to the BSA-Sepharose column in the same way. The unbound components, which were monospecific antibodies against digoxin (anti-digoxin), were collected. The bound antibodies were eluted with 0.2 M glycine-HCl (pH 2.5). The eluted fractions were neutralized immediately with 1 M Tris-HCl (pH 9.0) [17], pooled, and dialyzed. The flow rate for both columns was 1 mL/min. The antibody activity was evaluated by monospecific antibody ELISA and bispecific antibody ELISA.

### Demonstration of Fab-arm exchange *in vivo*

Anti-BSA antibodies were obtained from the BSA-immunized sera using a protein A column. Anti-digoxin antibodies were purified from the BSA-Sepharose column by the purification process described above. Female Balb/c nude mice (6–8 weeks) were purchased from Vital River Laboratory Animal Technology Co. Ltd (Beijing, China). Mice in the test groups were administered intravenous injections of the antibody (0.01 mg/each antibody/mouse), while the mice in the control group were administered intraperitoneally. Blood samples (75–100 µL) were drawn at scheduled time points, and centrifuged (10,000*g*, 5 min). Sera were collected and stored at −20°C until analysis.

Female New Zealand white rabbits (2.5–4.0 kg) were purchased from Beijing Xingwang nursery (Beijing, China). Rabbits were housed in a contained unit of the Animal Facility at Beijing Hospital (Beijing, China) and kept in cages with food and water. The administration procedures were almost the same as described above, except that the dose was 0.4 mg/each antibody/rabbit.

### Demonstration of Fab-arm exchange *in vitro*

A mixture of purified anti-BSA and anti-digoxin, at a final concentration of 100 µg/mL, was prepared and then reductive glutathione (GSH) was added to this mixture. The working concentration of GSH was 0.5 mM [4]. The mixture was incubated at 37°C for different intervals, and bispecific IgG was identified.

### Statistical analyses

The difference between female and male rabbits in bispecific antibody production was assessed using the paired *t*-test.

## Results

### Bispecific antibody could occur naturally in rabbits

#### Hapten-carrier immunization resulted in production of bispecific antibody

**Figure 1:**
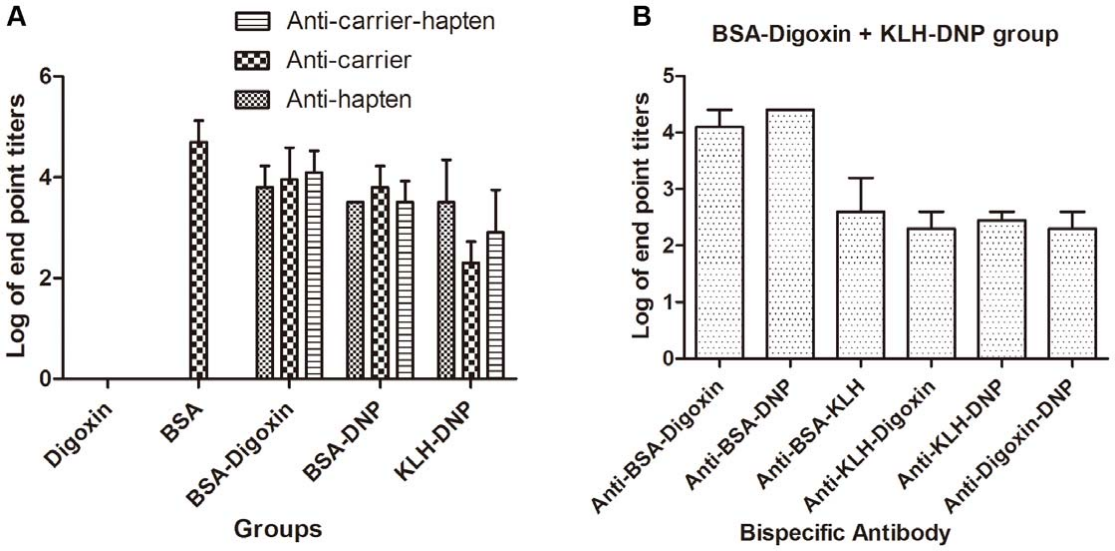
Antibody profiles in female rabbits immunized by different antigens. All female rabbits were bled 10 days after the last injection and sera were collected. Monospecific and bispecific antibody ELISAs were performed to define the antibody profiles. Columns represent the mean antibody titers of 2 female rabbits in each group. The error bars denote the standard deviation (SD). (A) Monospecific and bispecific antibody profiles in rabbits immunized with digoxin (*n* = 2), BSA (*n* = 2), BSA-digoxin (*n* = 2), BSA-DNP (*n* = 2), and KLH-DNP (*n* = 2). Rabbits immunized with conjugates produced a bispecific antibody against carrier and hapten in addition to monospecific antibodies to carriers or haptens. (B) Bispecific antibody profiles in rabbits immunized with a mixture of BSA-digoxin and KLH-DNP (*n* = 2). As many as 6 different types of bispecific antibodies were detectable in this group.

To investigate the antibody production patterns by different hapten-carrier conjugates, we allocated 24 New Zealand white rabbits into 6 groups according to the challenging antigens. Sera were obtained 10 days after the last injection and the antibody profiles were determined by monospecific and bispecific antibody ELISAs. Generally, 3 kinds of antibody were detectable in rabbits immunized with a single type of conjugate, including BSA-digoxin, BSA-DNP, or KLH-DNP. These were monospecific antibodies crosslinking haptens or carriers and bispecific antibodies crosslinking hapten and carrier (Fig. 1A). The maximum antibody titer was defined as a dilution giving an absorbance reading of 0.1 units over the blank signals, after subtracting background OD resulting from enzyme conjugates. The log of the sera maximum dilution was plotted on the Y-axis. In the rabbits immunized with a mixture of BSA-digoxin and KLH-DNP, as many as 6 different types of bispecific antibody were detected, including anti-BSA-digoxin, anti-BSA-DNP, anti-BSA-KLH, anti-KLH-digoxin, anti-KLH-DNP, and anti-digoxin-DNP antibodies (Fig. 1B). In the control groups, monospecific anti-BSA could be detected in the BSA group, whereas no antibody was observed in the digoxin group.

**Figure 2:**
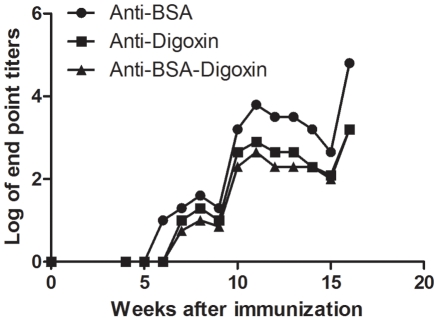
Longitude study of antibody production in a BSA-digoxin immunized rabbit. To monitor antibody production during immunization, sera were collected with 1-week intervals for 3 months after the first collection, which was conducted at 4^th^ week following the primary immunization. Monospecific and bispecific antibody ELISAs were performed and antibody titers were plotted against collecting time (weeks).

To monitor the production of bispecific antibodies during immunization, a longitudinal study was conducted in 1 rabbit immunized with BSA-digoxin. We began to collect the sera at the 4^th^ week after the primary immunization and with 1-week intervals for 3 months after that. The results showed that the bispecific antibody was first detected at the 7^th^ week. Though the antibody titers increased along with the immunization period, they were always lower than monospecific antibodies during the whole observation period (Fig. 2).

#### Female rabbits were more prone to produce bispecific antibodies

**Figure 3:**
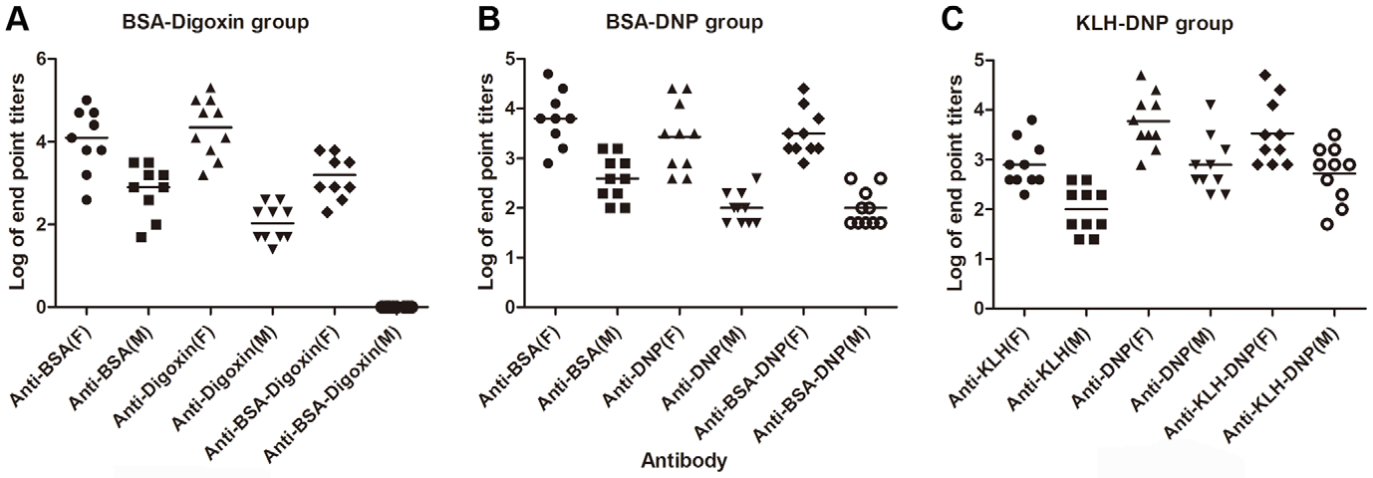
Comparison of antibody titers in female rabbits to male rabbits. Anti-carrier, anti-hapten, and bispecific antibody titers in the BSA-digoxin group (A), the BSA-DNP group (B), and the KLH-DNP (C) group were determined by monospecific and bispecific antibody ELISAs, respectively. Dots represent antibody titers of sera derived from the last 5 consecutive collections of female (*n* = 2, F) or male (*n* = 2, M) rabbits in each group. The lines denote the means of titers.

To test whether animal gender could influence the production of bispecific antibodies, 2 female and 2 male rabbits were included in each group. BSA-digoxin, BSA-DNP and KLH-DNP groups were involved in the comparison. Sera were collected consecutively in the last immunization period with 1-week intervals for 5 weeks. The differences in monospecific and bispecific antibody titers between the females and males were evaluated. Generally, antibody responses in female rabbits were collectively stronger than those in males. In the BSA-digoxin group, male rabbits did not produce any bispecific antibodies (Fig. 3A). However, in the BSA-DNP and KLH-DNP groups, bispecific antibodies were detected in male rabbits, but the titers were lower than those in females (Fig. 3B, 3C). There was a significant difference in the titers of the bispecific antibody between the 6 females and 6 males (*P*<0.01).

### Validation of natural bispecific antibody

#### Inhibition assays

**Figure 4:**
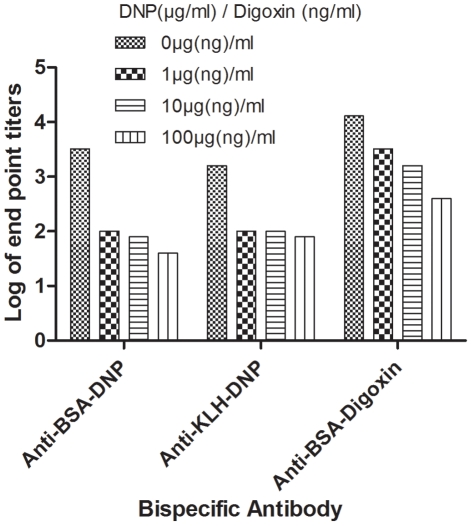
Inhibition assays for bispecific antibody. To determine the specificity of bispecific antibody, unlabeled digoxin (0, 1, 10, 100 ng/mL) or DNP (0, 1, 10, 100 µg/mL) were used as competitors with the HRP-labeled haptens in bispecific antibody ELISAs and the decreased antibody titers were plotted.

Inhibition assays were conducted to validate the specificity of bispecific antibodies. The results demonstrated that 1 µg/mL of L-lysine DNP resulted in 32 times (titers from 3200 to 100) and 16 times (1600 to 100) decreased titers in bispecific antibody assays for anti-BSA-DNP and anti-KLH-DNP respectively, compared with the original test in which no competitive haptens were added. However, haptens at 10 µg/mL and 100 µg/mL gave comparable results to 1 µg/mL. For anti-BSA-digoxin bispecific assay, a 4-fold inhibition (12800 to 3200) was achieved by 1 ng/mL of digoxin, an 8-fold (12800 to 1600) by 10 ng/mL, and a 32-fold (12800 to 400) by 100 ng/mL, compared with those in the original test (Fig. 4).

#### Bispecific antibodies were composed of IgG monomers

**Figure 5:**
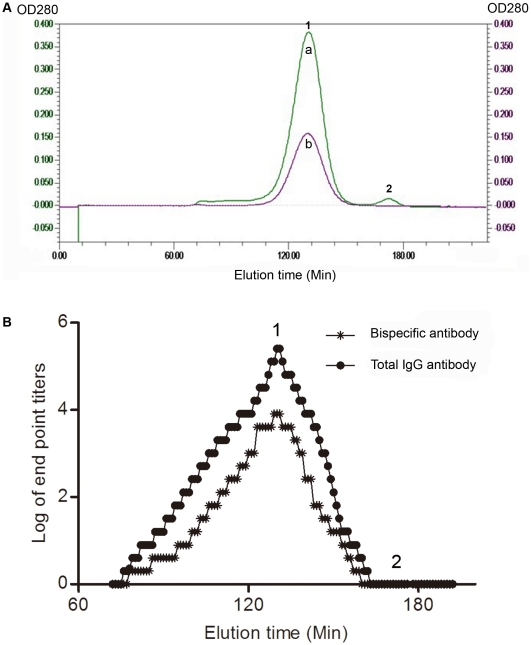
Size fractionation of anti-BSA-digoxin bispecific serum. (A) 200 µL of serum containing anti-BSA-digoxin bispecific antibody was fractionated on a Bio-Gel A1.5m column (a). Two distinct component peaks (peak 1 and peak 2) were obtained from the bispecific serum. A control, which consisted exclusively of IgG monomers, was separated on the same column with the same chromatographic conditions (b). The elution curves of bispecific serum and control overlapped after fractionation. (B) All 500 µL fractions of peak 1 and peak 2 were analyzed by bispecific antibody ELISA and their IgG nature was confirmed using a HRP conjugated goat anti-rabbit IgG antibody. The results were expressed as ELISA end point titers and plotted against the elution time.

To exclude the false bispecificity caused by antibody aggregates, serum (200 µL) containing anti-BSA-digoxin bispecific antibody and a control sample were fractionated on a Bio-Gel A1.5m column. Two distinct component peaks were obtained from the bispecific serum. The overlapped results indicated that the major one (peak 1) corresponded to the position derived from IgG monomers (Fig. 5A). Moreover, all the fractions of peaks 1 and 2 were analyzed by bispecific antibody ELISA and their IgG nature was confirmed using a HRP conjugated goat anti-rabbit IgG antibody. The results were expressed as the ELISA end point titers. Anti-BSA-digoxin bispecific antibodies and total IgG antibodies to BSA-digoxin were detected in the same fractions corresponding to the peak 1, whereas the fractions of the peak 2 did not display any antibody activities (Fig. 5B).

**Figure 6:**
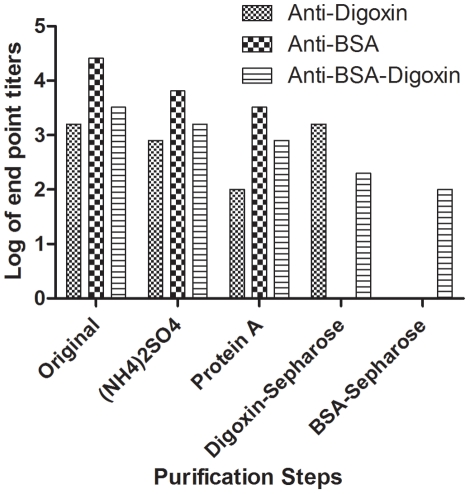
Purification of anti-BSA-digoxin bispecific antibodies. Anti-BSA-digoxin bispecific antibodies were purified from the serum using a series of purification methods. IgG fraction was obtained using 50% saturated ammonium sulfate precipitation, followed by passing through a protein A column. Bispecific antibody was further purified by affinity chromatography, using digoxin-Sepharose and BSA-Sepharose, after which a pure antibody fraction possessing bispecificity was obtained. Monospecific and bispecific antibody ELISAs were performed to monitor antibody titers during the entire purification process and the results were plotted.

To further validate the bispecific antibodies constituted a certain fraction of rabbit IgG antibody, we attempted to isolate the anti-BSA-digoxin bispecific antibodies from the sera. Antibody reactivity during the purification process was monitored using the monospecific and bispecific antibody ELISA. The IgG fraction obtained from the protein A column contained monospecific antibodies to digoxin (anti-digoxin), monospecific antibodies to BSA (anti-BSA), and anti-BSA-digoxin bispecific antibodies. Anti-BSA antibodies were depleted when passing through the digoxin-Sepharose column. Thus, the eluted fractions from the column retained anti-digoxin and anti-BSA-digoxin antibodies. Subsequently, BSA-Sepharose was used to remove anti-digoxin antibodies likewise, and the final collection represented bispecificity only (Fig. 6).

#### Bispecific antibody was not resulted from Fc-Fc interaction during detection

**Figure 7:**
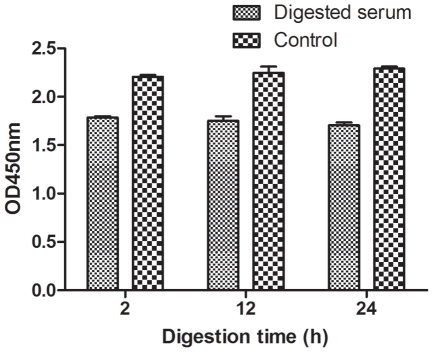
Pepsin digestion of bispecific serum. Anti-BSA-digoxin bispecific serum was subjected to pepsin digestion to exclude IgG Fc-Fc interactions, which could potentially result in false-positive signals in the bispecific analysis. After incubating the bispecific serum with 40 µg/mL of pepsin at 37°C for various intervals, the digestion reaction was stopped at 2, 12, and 24 h. Bispecific antibody was determined in digested samples and controls. Columns represent the mean optical densities at 450 nm (OD_450_) of duplicate measurements. Error bars denote the standard deviation (SD).

The Fc-Fc interaction between immunoglobulins is a potential source of artifacts in the measurement of bispecific antibodies [14], [18]. In order to exclude this possibility, we conducted a modified bispecific antibody ELISA in which the sera were treated with pepsin before the test. However, the bispecific antibody in our antiserum was not eliminated after pepsin digestion, although background binding of monospecific antibodies was observed (Fig. 7).

### Bispecific antibody could be generated *in vitro* and *in vivo*

**Figure 8:**
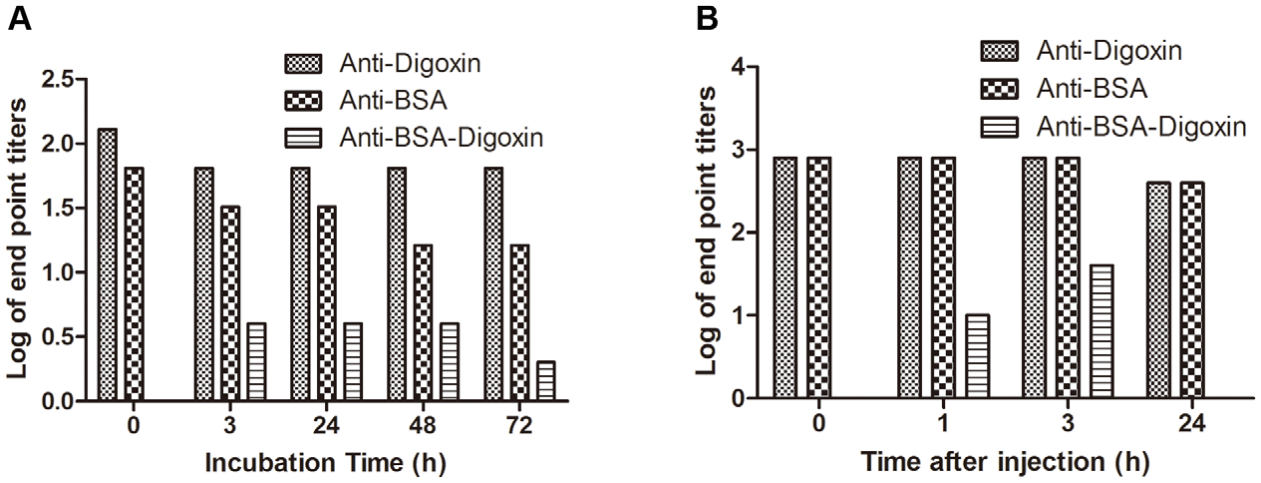
Demonstration of Fab-arm exchange *in vitro* and *in vivo*. (A) Two monospecific antibody preparations (anti-BSA and anti-digoxin) were mixed *in vitro* at the presence of 0.5 mM reductive GSH. (B) The mixture of anti-BSA and anti-digoxin was injected in a rabbit. Monospecific and bispecific antibody ELISAs were performed to monitor antibody titers at different time intervals after mixing anti-BSA with anti-digoxin *in vitro* or injecting the mixtures *in vivo*.

The generation of bispecific antibody by Fab-arm exchange was demonstrated *in vitro* and *in vivo*. After mixing 2 monospecific antibodies (anti-BSA and anti-digoxin) *in vitro* under a mild reductive environment, the bispecific antibodies against BSA and digoxin were initially observed at the 3^rd^ hour. It was maintained with incubation time, but decreased after 72 h (Fig. 8A). For in vivo test, no bispecific antibody was observed at any time point after injection in Balb/c nude (immunodeficient) mice (data not shown). However, in female New Zealand white rabbits, bispecific antibodies could be detected at the 1^st^ hour (titer of 10) and the 3^rd^ hour (titer of 40) after injection. However, the bispecific antibody became undetectable at the following time points (Fig. 8B).

## Discussion

In this report, we established an animal model for the production of a natural bispecific antibody by immunization with synthesized conjugates and conducted a series of studies to evaluate its characteristics.

Sera from rabbits immunized with BSA-digoxin, BSA-DNP, and KLH-DNP contained bispecific antibodies against the corresponding haptens and carriers, whereas such reactivity was not observed in control sera obtained in parallel. Inhibition assays confirmed the specificity of bispecific antibodies, because it could be suppressed to varying degrees by different concentrations of unlabeled haptens. However, full inhibition was not achieved in our experiments. Animals immunized with a carrier-hapten conjugate produce antibodies specific for 3 types of antigenic determinants: (1) the hapten determinant, (2) unaltered epitopes on the carrier protein, and (3) new epitopes formed by regions of both the hapten and carrier molecule in combination [19]. Thus, unlabeled haptens (unconjugated molecules) could not inhibit the HRP-labeled haptens (conjugated molecules) binding to bispecific antibodies when 1 Fab region was against the junction newly generated during conjugation. Naturally generated bispecific antibodies were recently described, in which human IgG4 from allergic patients who received immunotherapy with *Betula verrucosa* (Bet v 1) and Fel d 1 (major allergen from cat) manifested bispecificity [3]. We demonstrated that bispecific antibodies were also produced naturally in animal models.

There were 6 different types of bispecific antibodies present in sera of rabbits immunized with BSA-digoxin and KLH-DNP mixtures. These data suggest that the bispecific antibodies were readily produced by simultaneous stimulation with 2 distinct antigens, regardless of whether they were conjugated. This aspect was not adequately clarified in the study in relation to Bet v 1 and Fel d 1 [3].

We also investigated how gender contributed to the production of bispecific antibodies. In male rabbits (n = 6), the titers of bispecific antibodies were significantly lower than those in females (n = 6, p<0.01). It is generally believed that females generate a more vigorous immune response than males, but there is no consensus on the reason(s) underlying this difference [20]. The most frequent explanations suggest an immunosuppressive effect of testosterone in males, as demonstrated by immune enhancement after castration [21]–[22]. Also, women suffer more frequently and more severely from dysregulation of the immune system than men [23]–[24]. Our results indicated that sex hormones might play a role in bispecific antibody production. However, larger animal populations and other researches are necessary to further explore this.

Longitudinal observations in rabbits revealed that the initial levels of bispecific antibodies were low, and the titers increased during immunization periods, along with monospecific antibodies. The bispecific antibody appeared 2 weeks after the appearance of antibodies against BSA and at nearly the same time as antibodies against digoxin. This prompted us to reconsider the origin of the bispecific antibodies, specifically, whether they were induced by exchanges between 2 monospecific antibodies (anti-BSA and anti-digoxin). To examine this, we conducted *in vitro* and *in vivo* experiments as described previously [4]. We demonstrated that Fab-arm exchanges occurred *in vitro* in a mild reductive environment (GSH). However, *in vivo*, we reproduced this only in rabbits and failed to demonstrate Fab-arm exchange in mice. We suggest that a homologous environment may facilitate Fab-arm exchanges. About 80% of mouse genes have a single identifiable human orthologue, and fewer than 1% of mouse genes appear to be truly unique [25]. Thus, the homologous internal environment may be a requirement for observable human Fab-arm exchanges in mice. Presumably, the rabbit antibodies share too little homology with mice.

As determined by size fractionation and rabbit IgG specific ELISA, the bispecific antibodies were of the IgG isotype and existed as IgG monomers. Human IgG consists of 4 subclasses (1–4) that can be recognized by antigen differences in their heavy chains. Human IgG4 has been reported as a type of native bispecifc antibody [3]–[4]. However, in rabbits, only a single IgG subclass has been identified [26]. An ELISA method was established to detect rabbit bispecific antibodies by means of various antigen pairs. Nonetheless, false-positive results caused by antibody aggregates or Fc-Fc interaction could have been involved in the detection. However, the bispecific antibody was validated not only by size chromatography, by comparison with the control, but also by pepsin digestion. Additionally, we obtained the fraction of bispecific IgG after serial purification processes.

It has been known for more than half a century that in most immune sera a population of antibodies exists that is unable to form precipitates with the antigen [27]. Objectively, bispecific antibody is another form of non-precipitating antibody. Many explanations have been proposed for the inability of non-precipitating antibodies to precipitate with antigens. Steric interference between 2 Fabs has been mentioned as a possible mechanism, but the most likely explanation is structure asymmetry: 1 Fab being different from the other. In principle, this could be achieved in different ways, such as lack of allelic exclusion, post-translational modification, and post-secretional modification [28]. In the case of IgG4, Aalberse et al. favored the third explanation. They hypothesized that IgG4 was secreted by the plasma cells as a normal symmetry antibody, but subsequently interacts with another IgG molecule and Fab-arm exchange occurs. This process results in IgG4 molecules with 2 distinct antigen-binding sites [29]. Likewise, this hypothesis is also applicable to rabbit bispecific antibodies, because we demonstrated Fab-arm exchanges *in vitro* and *in vivo*. The Fab-arm exchange has been suggested to be a novel type of modification for generating anti-inflammatory activity [4]. A comprehensive examination of the significance of this modification needs further studies.

In summary, we established an animal model for the production of a natural bispecific antibody and conducted studies to examine its characteristics. Human bispecific IgG4 and bispecific antibodies from animal models represent a group of antibody products resulting from simultaneous stimulation by 2 distinct antigens. This prompted us to consider similar statuses relevant to human diseases or therapies. As our results indicated, bispecific antibodies were produced readily when 2 distinct antigens were used for simultaneous immunization, and sex hormones may potentially influence its production. On the other hand, the novel protein modification mechanism challenges the commonly accepted one antibody–one antigen paradigm and redefines our thinking about the role and application of naturally produced bispecific antibodies.
